# Incidence and Extent of Graft Extrusion following Meniscus Allograft Transplantation

**DOI:** 10.1155/2018/5251910

**Published:** 2018-03-25

**Authors:** Dae-Hee Lee

**Affiliations:** Department of Orthopaedic Surgery, Samsung Medical Center, Sungkyunkwan University School of Medicine, Seoul, Republic of Korea

## Abstract

**Background:**

The incidence and extent of graft extrusion after meniscus allograft transplantation (MAT) may differ in patients undergoing medial and lateral meniscus transplantation due to the use of different surgical techniques. This meta-analysis was therefore designed to quantify the extent and incidence of graft extrusion after meniscus allograft transplantation.

**Methods:**

Following preferred reporting items for systematic reviews and meta-analyses (PRISMA) guidelines, all studies reporting absolute extrusion, relative percentage of extrusion (RPE), or frequency of major extrusions (>3 mm) on magnetic resonance imaging in patients undergoing medial or lateral MAT were included.

**Results:**

The pooled mean absolute extrusion following all MATs was 3.15 mm but was significantly greater following medial than lateral MAT (3.26 versus 3.01 mm; *p* = 0.001). The pooled mean RPE following all MATs was 32.79% and was significantly greater after medial than lateral MAT (32.69% versus 28.81%; *p* < 0.001). The pooled mean proportion of knees with major (>3 mm) extrusion was 53% (95% CI: 49% to 58%) and was significantly greater after medial than lateral MAT (61% versus 39%; *p* < 0.001).

**Conclusion:**

Mean graft extrusion after arthroscopic assisted MAT using bony fixation was 3.2 mm, with major graft extrusion > 3 mm occurring in about 50% of transplanted menisci. Graft extrusion was significantly greater after medial than lateral MAT.

## 1. Introduction

Meniscus allograft transplantation (MAT) is an established surgical treatment for patients with subtotal or total meniscectomized knees [1, 2], providing both pain relief and functional improvement [3–5]. In addition, MAT has been reported to alleviate the progression of osteoarthritis [6], although it is not as effective as the native meniscus. The postoperative status of transplanted meniscus allografts has been evaluated noninvasively using methods such as magnetic resonance imaging (MRI). Postoperative MRI following MAT, however, has shown extrusion of the meniscus allograft, similar to findings in osteoarthritic knees with meniscus tears [7]. The incidence and extent of extrusion after MAT, however, remain undetermined. Moreover, the precise causes of graft extrusion after MAT remain unclear. Although studies have indicated that graft extrusion may be due to surgical technique, especially the incorrect determination of the positions of the anterior and posterior horns of the transplanted meniscus, the obvious errors in surgical technique were not always found in the cases of extrusion following MAT.

Many MATs are currently performed using arthroscopic assisted bony fixation techniques. The bone plug technique has been used for medial side MATs and the keyhole technique for lateral side MATs, with both techniques involving bony fixation of the anterior and posterior horns of the meniscus [8]. Inasmuch as surgical techniques differ between medial and lateral MAT, so may the incidence and extent of graft extrusion. In addition, extrusion could have already been present prior to surgery in the medial and lateral menisci.

This meta-analysis was therefore designed to quantify the incidence and extent of graft extrusion after MAT. This meta-analysis hypothesized that graft extrusion occurs after both medial and lateral MAT, whereas the incidence and extent graft extrusion may differ after medial and lateral MAT.

## 2. Materials and Methods

### 2.1. Data and Literature Sources

The study design was based on Cochrane Review Methods. Multiple comprehensive databases, including MEDLINE, EMBASE, and the Cochrane Library, were searched for studies that compared graft extrusion on postoperative MRI in patients who underwent medial and lateral MAT. There were no restrictions on language or year of publication. Search terms used in the title, abstract, MeSH, and keywords fields included “Meniscus” [tiab] OR “Transplantation” [tiab] OR “Extrusion” [tiab] AND “Knee” [tiab], and “Menisci” [MeSH] OR “Transplantation” OR “Organ transplantation” [MeSH]. After the initial electronic search, relevant articles and their bibliographies were searched manually. Articles identified were assessed individually for inclusion.

### 2.2. Study Selection

Study inclusion was decided independently by two reviewers, based on the predefined selection criteria. Titles and abstracts were read; if suitability could not be determined, the full article was evaluated. Studies were included in the meta-analysis if (1) they included patients who underwent arthroscopic assisted medial and/or lateral MAT with bone plug or bone block fixation and who underwent postoperative MRI for evaluation of graft extrusion; and (2) they reported at least one parameter related to extrusion, including absolute extrusion (mm), relative percentage of extrusion (RPE, %), or proportion of subjects with major extrusion. Absolute extrusion was defined as the distance between the outer edge of the articular cartilage of the tibial plateau and the meniscal outer edge. RPE was defined as the percentage width of an extruded meniscus relative to the width of the entire meniscus. Major extrusion was defined as extrusion > 3 mm after MAT. Studies were also included if they (3) fully reported the numbers of knees that underwent MAT, the means and standard deviations of absolute extrusion and RPE, and the proportion of all patients who exhibited major extrusion. To reduce heterogeneity due to differences in surgical technique, studies were excluded if they evaluated an open rather than an arthroscopic approach or if soft tissue fixation rather than a bone plug or bone block fixation was utilized.

### 2.3. Data Extraction

Two reviewers independently recorded data from each study using a predefined data extraction form. If any disagreement could not be resolved by discussion, the data were reviewed by a third reviewer. Variables associated with surgical method included method of fixation (soft tissue or bony fixation), and, if bony fixation was used, whether it was performed using a bone plug or keyhole methods. Also recorded were sample size; the means and standard deviations of absolute extrusion and RPE; and the proportion of patients with major (>3 mm) extrusions. Some of the evaluated studies did not report the absolute numbers of knees that underwent medial and/or lateral MAT individually but reported extrusion data on total numbers of knees; these studies were included, with data from all knees combined. If these variables were not mentioned in the articles, the study authors were contacted by email to request these data.

### 2.4. Assessment of Methodological Quality

Two reviewers independently assessed the methodological quality of each study using the Newcastle-Ottawa Scale [9], as recommended by the Cochrane Non-Randomized Studies Methods Working Group. In this study, the Newcastle-Ottawa Scale's star system, which awards stars depending on the level of bias, was adjusted to a scale that included only low (one star), high, and unclear bias. Each study was judged on three criteria: the selection of the study groups, the comparability of the groups, and the determination of either exposure or outcome of interest for case-control or cohort studies. Any unresolved disagreements between reviewers were resolved by consensus or by consultation with a third investigator.

### 2.5. Statistical Analysis

The main outcomes of the meta-analysis were the mean differences in absolute extrusion and RPE and the proportion of subjects with major extrusion, in groups of patients that had undergone medial and/or lateral MAT. Odds ratios (OR) and 95% confidence intervals (CIs) were calculated for binary outcomes, whereas mean differences and 95% CIs were calculated for continuous outcomes. Heterogeneity was determined by estimating the proportion of between-study inconsistencies due to actual differences between studies, rather than differences due to random error or chance, using the *I*^2^ statistic, with values of 25%, 50%, and 75% defined as low, moderate, and high, respectively. Publication bias was also assessed using funnel plots and Egger's test. All statistical analyses were performed using RevMan version 5.2 and Stata/MP 13.0. A *p* value < 0.05 was considered significant.

## 3. Results

### 3.1. Identification of Studies

**Figure 1:**
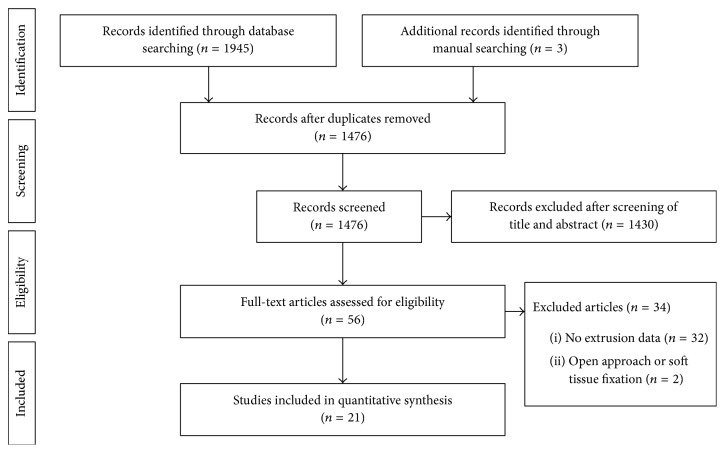
PRISMA (preferred reporting items for systematic reviews and meta-analyses) flow diagram of the identification and selection of the studies included in this meta-analysis.

The details of study identification, inclusion, and exclusion are illustrated in Figure 1. An electronic search yielded 794 studies in PubMed (MEDLINE), 1107 in EMBASE, and 44 in the Cochrane Library. Three additional publications were identified through manual searching. After removing 469 duplicates, 1476 studies remained; of these, 1430 were excluded based on reading of the titles and abstracts, 32 were excluded to the lack of extrusion data, and two were excluded because they used an open approach or soft tissue fixation. After applying these criteria, 21 studies [4, 9–20, 22–26, 28–30] were finally included in this meta-analysis.

### 3.2. Study Characteristics and Patient Populations

Of the 21 studies included in the meta-analysis, 10 reported meniscus extrusion parameters in medial and lateral MATs separately and six reported meniscus extrusion parameters of medial and lateral MATs altogether. Five studies reported meniscus extrusion parameters in lateral MAT, with only one reporting meniscus extrusion parameters in medial MAT. All 21 studies retrospectively investigated absolute and/or relative extrusion and/or the proportion of knees with major extrusion. Six studies reported absolute extrusion, RPE, and the proportion with major extrusion; six reported absolute extrusion and RPE; and two reported absolute extrusion and proportion of major extrusion. Five studies reported absolute extrusion alone, one reported the proportion with major extrusion, and one reported RPE alone (Table 1).

### 3.3. Quality and Publication Bias of the Included Studies

**Figure 2:**
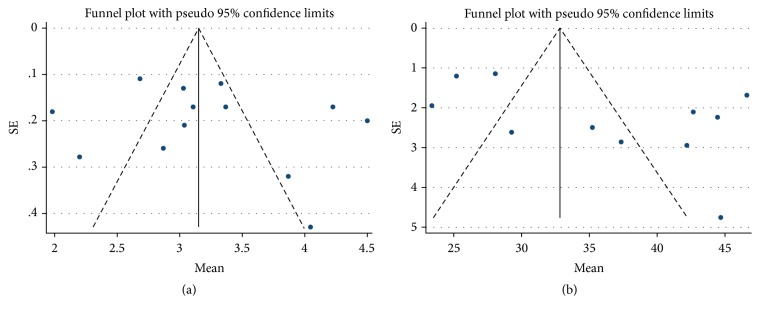
Funnel plots showing relatively symmetrical absolute extrusion (a) and relative percentages of extrusion (RPE, (b)) in transplanted meniscus, merging extrusion after medial and lateral MATs.

All 21 studies included in this meta-analysis had a low risk of selection bias, whereas none assessed possible confounding factors. A shorter time interval between surgery and the performance of MRI was associated with a lower risk of bias, although graft extrusion was not likely to increase over time. Studies were considered at low risk of bias if postoperative MRI was performed within 1 year after surgery. The risk of bias for the studies included in this meta-analysis is summarized in Table 2. Funnel plots showed that mean absolute extrusion and RPE after medial and lateral MATs were relatively symmetric, indicating lack of publication biases among the included studies, both in assessing absolute extrusion (Figure 2(a)) and RPE (Figure 2(b)). Egger's test also revealed no significant publication biases in both absolute extrusion (*p* = 0.515) and RPE (*p* = 0.094).

### 3.4. Absolute Extrusion

**Figure 3:**
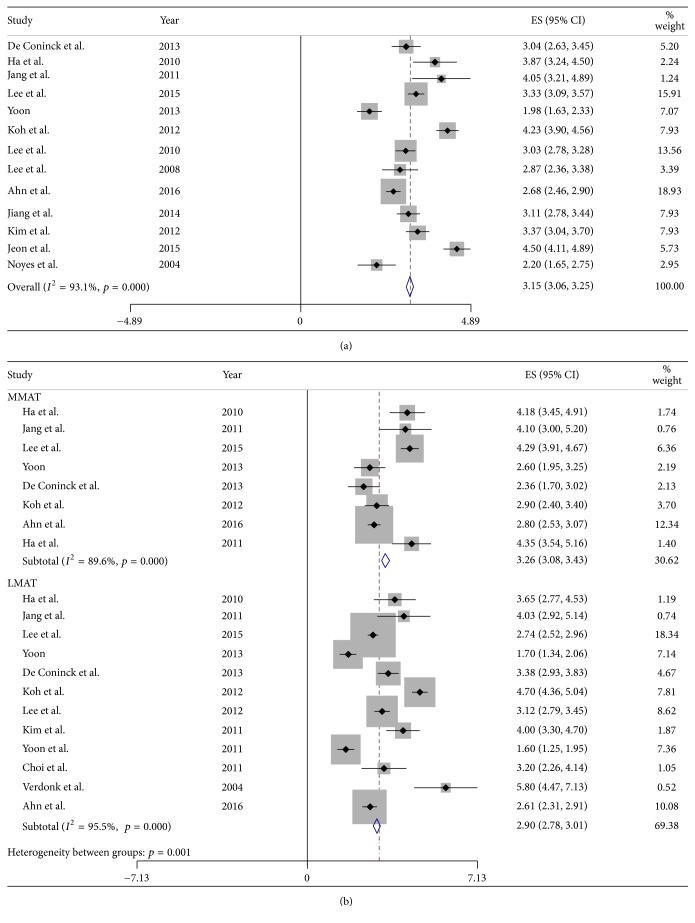
Forest plots showing absolute extrusion in merged medial and lateral MATs (a) and in each individually (b).

Of the 21 studies, 13 reported the mean absolute extrusion of the transplanted meniscus, merging extrusion after medial and lateral MATs. Eight studies reported mean absolute extrusion after medial MAT, and 12 reported mean absolute extrusion after lateral MAT. The pooled mean absolute extrusion following all MATs, including medial and lateral MATs, was 3.15 mm (95% CI: 3.06 to 3.25 mm; Figure 3(a)) and 3.26 mm (95% CI: 3.08 to 3.43 mm) after medial and 2.90 mm (95% CI: 2.78 to 3.01 mm; Figure 3(b)) after lateral MAT, indicating that graft extrusion was 0.25 mm greater after medial than after lateral MAT (*p* = 0.001).

### 3.5. Relative Percentage of Extrusion (RPE)

**Figure 4:**
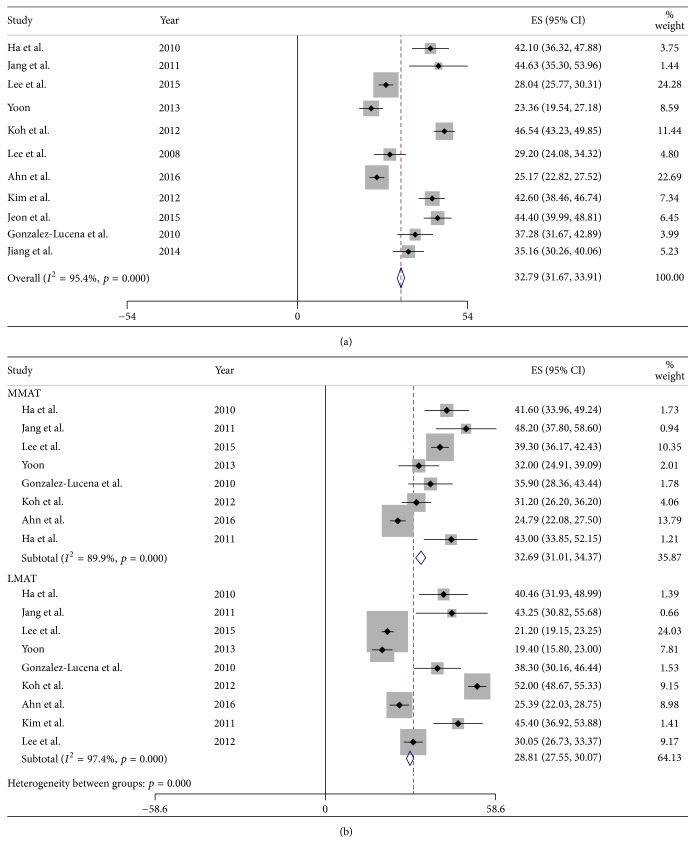
Forest plots showing the relative percentage of extrusion (RPE) in merged medial and lateral MATs (a) and in each individually (b).

Of the 21 studies, 11 reported mean relative percentage of extrusion (RPE) for combined medial and lateral MATs. Eight studies reported mean RPE after medial MAT, and nine reported median RPE after lateral MAT. The pooled mean RPE after all MATs, including medial and lateral MATs, was 32.79% (95% CI: 31.67% to 33.91%; Figure 4(a)) and 32.69% (95% CI: 31.01% to 34.37%) after medial and 28.81% (95% CI: 27.55% to 30.07%; Figure 4(b)) after lateral MAT. Thus, mean RPE was 3.88% greater after medial than after lateral MAT (*p* < 0.001).

### 3.6. Proportion of Major Extrusion

**Figure 5:**
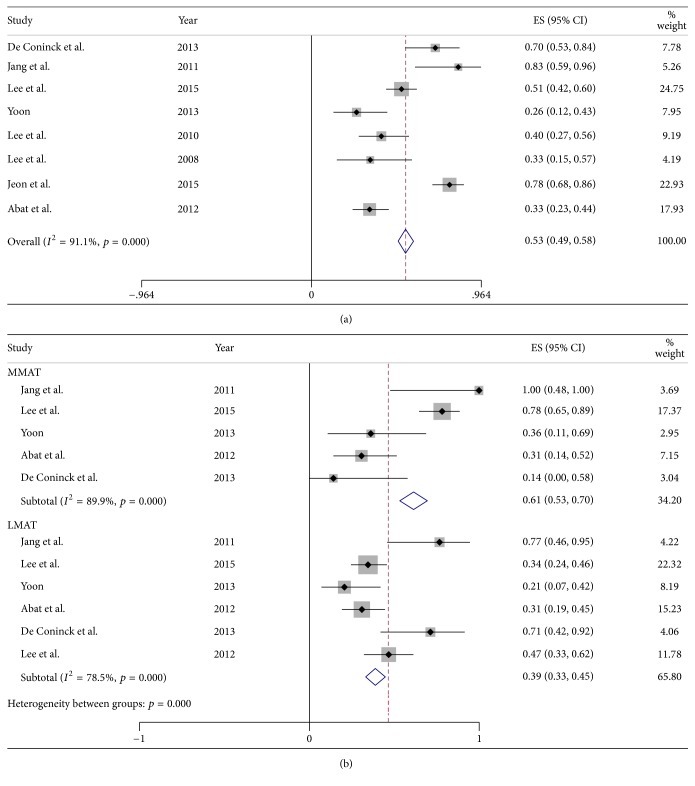
Forest plots showing the proportions of subjects with major graft extrusion (>3 mm) in merged medial and lateral MATs (a) and in each individually (b).

Eight studies reported the proportion of knees with major extrusion (>3 mm) for combined medial and lateral MATs. Five studies reported the proportion with major extrusion after medial MATs, and six studies reported the proportion of major extrusion after lateral MATs. The pooled mean proportion of all knees with major extrusion was 53% (95% CI: 49% to 58%; Figure 5(a)) and 61% (95% CI: 53% to 70%) after medial and 39% (95% CI: 33% to 45%; Figure 5(b)) after lateral MATs. These findings showed that the proportion of knees with major extrusion was 22% greater after medial than after lateral MAT (*p* < 0.001, Table 3).

## 4. Discussion

The current meta-analysis demonstrated that graft extrusion after MAT could not be completely avoided, not only on the medial but also on the lateral side. Mean graft extrusion was approximately 3 mm, equal to about one-third of the width of the meniscus. Although the precise causes of graft extrusion remain undetermined, several possible causes have been suggested [3, 26]. Two sets of factors may be associated with meniscus extrusion; one set is inevitable or difficult to reduce and is not associated with surgical technique, whereas the other can be reduced by a more accurate surgical technique. These latter factors have been associated with surgical error in determining the locations of the anterior and posterior horns of the transplanted meniscus. Normal anatomic features, such as the meniscotibial ligament and popliteomeniscal fascicle, are inherent limitations of meniscus allograft transplantation [2]. Both of these structures function to anchor the meniscus, reducing the risk of graft extrusion. In addition, most knees undergoing MAT have lost their normal joint geometry, due to osteoarthritic changes, including flattening of the femoral condyle, concavity of the tibial plateau, and osteophytes. These deformations in the joint space environment may interrupt the anatomical seating of the transplanted meniscus, with graft extrusion due to a size mismatch between the meniscus allograft and the deformed joint space. As oversized grafts almost guarantee extrusion [3], preoperatively inaccurate graft sizing can cause graft extrusion. There is no general consensus on the optimal radiographic imaging tool for sizing, as calculations based on plain radiographs and MRI use different constant values for tibial plateau width and length.

Surgery during MAT, especially lateral MAT [12, 23, 26], may result in graft extrusion on postoperative MRI. Studies of graft extrusion on postoperative MRI have emphasized the importance of placing the center of the bone bridge as close as possible to the middle of the tibial plateau [12, 26]. Moreover, reductions in the axial trough angle may reduce the risk of graft extrusion following lateral MATs [23]. The bony trough starting point in lateral MAT should not be made too laterally, as the resulting increase in the distance from the center of the tibial plateau to the bony trough and the obliquity of the bony trough on the axial plane and incorrect horn positioning can lead to graft extrusion.

Graft extrusion may also be due to overtensioning of the meniscal suture during surgery. One study recommended that, after determining the appropriate thread tension by pulling the thread out under arthroscopic visualization, each tie should be performed with appropriate tension under arthroscopic visualization [2].

This meta-analysis also showed that the absolute and relative amounts of extrusion were greater and that the incidence of major extrusion (>3 mm) was higher, after medial MAT than after lateral MAT. Both the increases in graft extrusion and the incidence of major extrusion may be due to the drawbacks of the arthroscopically assisted bone plug technique for medial MAT. In this procedure, the positions of the anterior and posterior horns were determined separately. While the posterior horn position was determined under arthroscopic guidance, the anterior horn position was determined with the naked eye [31]. Positioning the tunnel for the bone plug at an accurate anatomical horn position is difficult, as soft tissue such as patellar fat pads can obstruct the view of the anterior horn. Furthermore, determining the tunnel position for bone plugs of the posterior horn was also difficult, because arthroscopic visualization of the medial meniscus posterior horn from the anterolateral routine portal was made difficult by the narrow joint space. Despite the use of posteromedial viewing portal, determining the posterior horn location is complicated by the absence of a definitive bony or soft tissue landmark. These surgical difficulties in determining horn positions may have resulted in erroneous positioning of the anterior or posterior horn during medial MAT. In the arthroscopically assisted keyhole technique for lateral MAT; however, the anterior and posterior horn positions were determined by the location of the bony trough, allowing reaming along the inserted guide pin. Surgeons tend to avoid using a too lateral starting point for the guide pin, because a too laterally positioned guide pin could increase graft extrusion through a lateral trough position and increased axial trough angle on the axial plane [12, 23]. Additionally, the greater physiologic extrusion of normal medial menisci compared to normal lateral menisci has been found to be due to a greater overstuffing effect of the former, resulting from the greater thickness of the medial than of the lateral meniscus [32]. Likewise, the higher incidence of major extrusion following medial than lateral MAT may have been due to the greater overstuffing of the inherently thicker medial meniscus allograft into a restricted joint space.

This study had several limitations. All the studies included in this meta-analysis were observational comparison studies, which are prone to both systematic and random error, suggesting inherent heterogeneity due to uncontrolled bias [33]. Additionally, slight differences in other factors that could affect graft extrusion may make the included studies heterogeneous. Size mismatches between allografts and recipients, difference in details of surgical techniques, and different time points of postoperative MRI may have affected postoperative graft extrusion, which may explain, at least in part, the heterogeneity of the results of this meta-analysis. However, efforts were made to minimize the heterogeneity resulting from different surgical techniques by only including studies using arthroscopic assisted approaches and bone plugs or fixation blockers and by excluding the studies using an open approach or soft tissue fixation. This study did not consider the native meniscus extrusion prior to surgery, which could affect the magnitude of meniscus allograft transplantation. However, there was a lack of studies that reported preoperative native meniscus extrusion, and a recent study [34] showed that extrusion of the postoperative lateral meniscus allograft was not associated with preoperative extrusion of the lateral native meniscus. Finally, the effect of meniscus allograft extrusion on clinical outcomes was not elucidated in the current study. Although a midterm follow-up study reported that graft extrusion did not appear to affect 5-year clinical or radiological outcomes [22], prospective studies with robust designs and sufficient sample sizes are warranted in the future.

In conclusion, mean graft extrusion after arthroscopic assisted MAT using bony fixation was 3.2 mm, with approximately one-half of transplanted menisci showing evidence of major graft extrusion greater than 3 mm meniscus. In addition, graft extrusion was greater after medial than lateral MAT.

## Figures and Tables

**Table 1:** Characteristics of the studies included in the meta-analysis.

Authors	Year	Study type	Sample size	Measured parameters	Mean time from surgery to MRI
Abat et al. [10]	2012	RCS	MMAT (26), LMAT (29)	PME	3.5 years
Ahn et al. [11]	2016	RCS	MMAT (23), LMAT (41)	AE, RPE	1 year
Choi et al. [12]	2011	RCS	LMAT (23)	AE	6 months
De Coninck et al. [13]	2013	RCS	MMAT (7), LMAT (14)	AE, PME	1 year
Gonzalez-Lucena et al. [14]	2010	RCS	MMAT (14), LMAT (19)	RPE	5 years
Ha et al. [7]	2010	RCS	MMAT (15), LMAT (14)	AE, RPE	2 years
Ha et al. [15]	2011	RCS	MMAT (18)	AE, RPE	At least 1 year
Jang et al. [16]	2011	RCS	MMAT (5), LMAT (13)	AE, RPE, PME	2.5 years
Jeon et al. [17]	2015	RCS	Without distinction of MMAT or LMAT (88)	AE, RPE, PME	1 year
Jiang et al. [18]	2014	RCS	Without distinction of MMAT or LMAT (18)	AE	4.2 years
Kim et al. [19]	2011	RCS	LMAT (28)	AE, RPE	3.4 years
Kim et al. [20]	2012	RCS	Without distinction of MMAT or LMAT (108)	AE, RPE	2.4 years
Koh et al. [21]	2012	RCS	MMAT (26), LMAT (73)	AE, RPE	2 years
Lee et al. [2]	2008	RCS	Without distinction of MMAT or LMAT (21)	AE, RPE, PME	6 weeks
Lee et al. [22]	2010	RCS	Without distinction of MMAT or LMAT (43)	AE, PME	1 year
Lee et al. [23]	2012	RCS	LMAT (49)	AE, RPE, PME	Immediate postoperative
Lee et al. [24]	2015	RCS	MMAT (51), LMAT (84)	AE, RPE, PME	Immediate postoperative
Noyes et al. [9]	2004	RCS	Without distinction of MMAT or LMAT (29)	AE	3 years
Verdonk et al. [25]	2004	RCS	LMAT (17)	AE	2 years
Yoon et al. [26]	2011	RCS	LMAT (11)	AE	1 year
Yoon et al. [27]	2014	RCS	MMAT (11), LMAT (24)	AE, RPE, PME	1 year

M(L)MAT, medial (lateral) meniscus allograft transplantation; RCS, retrospective comparison study; AE, absolute extrusion; RPE, relative percentage of extrusion; PME, proportion of major extrusion.

**Table 2:** Risk of bias summary: review authors' judgments about the risk of bias item for each included study.

Authors	Year	Representativeness of the cases	Selection of control	Ascertainment of exposure	Outcome of interest not present at study onset	Comparability of cohorts	Control for any additional factor	Assessment of outcome	Sufficient follow-up	Adequacy of follow-up
Abat et al. [10]	2012	−	−	−	−	+	+	+	+	−
Ahn et al. [11]	2016	−	−	−	−	+	+	−	−	−
Choi et al. [12]	2011	−	−	−	−	+	+	+	−	−
De Coninck et al. [13]	2013	−	−	−	−	+	+	+	−	−
Gonzalez-Lucena et al. [14]	2010	−	−	−	−	+	+	+	+	−
Ha et al. [7]	2010	−	−	−	−	−	+	+	+	−
Ha et al. [15]	2011	−	−	−	−	−	+	+	+	−
Jang et al. [16]	2011	−	−	−	−	−	+	+	+	−
Jeon et al. [17]	2015	−	−	−	−	+	+	−	−	−
Jiang et al. [18]	2014	−	−	−	−	+	+	+	+	−
Kim et al. [19]	2011	−	−	−	−	+	+	+	+	−
Kim et al. [20]	2012	−	−	−	−	+	+	−	+	−
Koh et al. [21]	2012	−	−	−	−	+	+	+	+	−
Lee et al. [2]	2008	−	−	−	−	+	+	−	−	−
Lee et al. [22]	2010	−	−	−	−	+	+	+	−	−
Lee et al. [23]	2012	−	−	−	−	+	+	+	−	−
Lee et al. [24]	2015	−	−	−	−	+	+	+	−	−
Noyes et al. [9]	2004	−	−	−	−	−	+	+	+	−
Verdonk et al. [25]	2004	−	−	−	−	−	+	+	+	−
Yoon et al. [3]	2011	−	−	−	−	−	+	+	−	−
Yoon et al. [27]	2014	−	−	−	−	−	+	+	−	−

^−^Low risk of bias;  ^+^high risk of bias.

**Table 3:** Pooled mean absolute and relative extrusions; percentage of major graft extrusion in medial and lateral meniscus transplantation and total (medial and lateral side) meniscus transplantation.

	MMAT Pooled mean (95% CI)	LMAT Pooled mean (95% CI)	*p* value MMAT versus LMAT	Total (MM + LM) MAT
				Pooled mean (95% CI)
Absolute extrusion (mm)	3.26 (3.08 to 3.43)	2.90 (2.78 to 3.01)	0.001	3.15 (3.06 to 3.25)
Relative percentage of extrusion (%)	32.69 (31.01 to 34.37)	28.81 (27.55 to 30.07)	<0.001	32.79 (31.67 to 33.91)
Percentage of major extrusion (>3 mm)	61 (53 to 70)	39 (33 to 45)	<0.001	53 (49 to 58)
